# Supplementary figures and images for: A role for the immune system-released activating agent (ISRAA) in the ontogenetic development of brain astrocytes
Source: PLoS One. 2021 May 10;16(5):e0248455. doi: 10.1371/journal.pone.0248455 (PMC8109834; doi:10.1371/journal.pone.0248455)

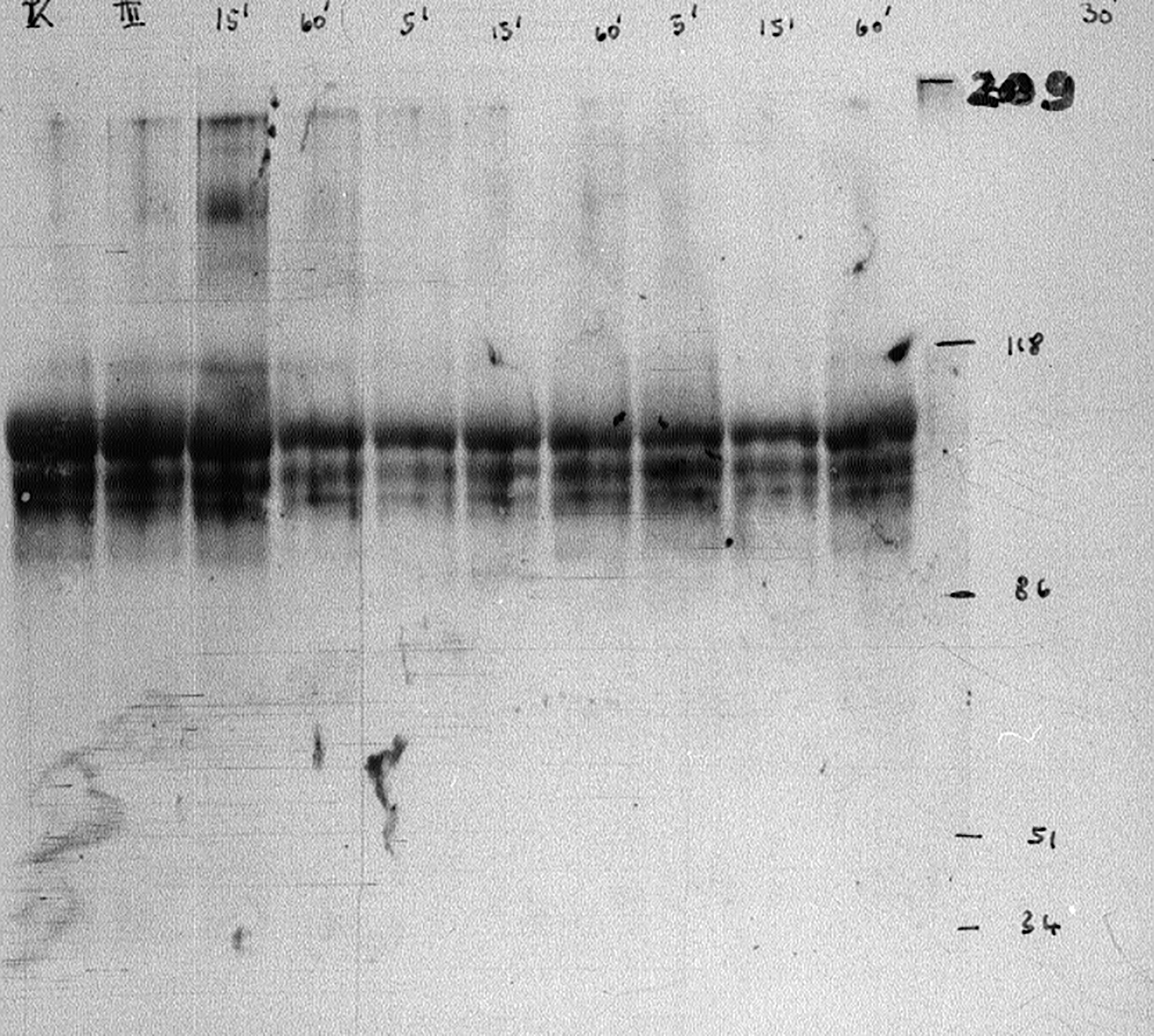

Supplement: S1 Fig — Cell lysates were prepared 5min, 15min and 60min after ISRAA stimulation. K in the 1st lane (non-stimulated control lysates). ISRAA stimulation (2nd, 3rd and 4th lanes) resulted in a strong and fast rise in tyrosine phosphorylation, which reached its highest point by 15min and declined to the control level at 60min. An irrelevant antibody was used to control the specificity of the phosphorylated protein (5th, 6th and 7th lanes). Tyrphostin A47 (a tyrosine kinase specific inhibitor) blocked the ISRAA induced protein kinase at the various detection times (8th, 9th and 10th lanes). (TIF) [file pone.0248455.s001.tif]
